# Draft Genome Sequences of a Putative Prokaryotic Consortium (IPPAS B-1204) Consisting of a Cyanobacterium (*Leptolyngbya* sp.) and an Alphaproteobacterium (*Porphyrobacter* sp.)

**DOI:** 10.1128/MRA.01637-18

**Published:** 2019-04-11

**Authors:** Kirill S. Mironov, Pavel A. Leusenko, Vera V. Ustinova, Kenzhegul Bolatkhan, Bolatkhan K. Zayadan, Elena V. Kupriyanova, Maria Shumskaya, Maria A. Sinetova, Dmitry A. Los

**Affiliations:** a K. A. Timiryazev Institute of Plant Physiology, Russian Academy of Sciences, Moscow, Russia; b Microbiology Department, Central Tuberculosis Research Institute, Moscow, Russia; c Department of Biotechnology, Faculty of Biology and Biotechnology, Al-Farabi Kazakh National University, Almaty, Kazakhstan; d School of Natural Sciences–Biology, Kean University, Union, New Jersey, USA; University of California, Riverside

## Abstract

A new presumably simple consortium of a *Leptolyngbya* sp. and a *Porphyrobacter* sp. was isolated from Tolbo Lake in Mongolia. The draft genome sequences of both species are reported. The consortium has been deposited in the Collection of Microalgae and Cyanobacteria of the Institute of Plant Physiology, Moscow, Russia, under the accession number IPPAS B-1204.

## Abstract (precis)

A new presumably simple consortium of a *Leptolyngbya* sp. and a *Porphyrobacter* sp. was isolated from Tolbo Lake in Mongolia. The draft genome sequences of both species are reported.

## ANNOUNCEMENT

A consortium consisting of two species, a *Leptolyngbya* sp. and a *Porphyrobacter* sp., was isolated from Tolbo Lake, an alpine lake of glacial origin (West Mongolia, 48°32′56ʺN 90°03′03ʺE, 2,079 meters above sea level). The consortium was deposited in the Collection of Microalgae and Cyanobacteria of the Institute of Plant Physiology of the Russian Academy of Sciences (IPPAS) under the accession number IPPAS B-1204 (http://www.cellreg.org/Catalog/Catalog%20NEW/IPPAS%20B-1204.html).

The consortium was grown photoautotrophically in BG-11 medium under 50 µmol m^−2^ · s^−1^ photons of cool white light aerated by air enriched with 1.5% CO_2_ (vol/vol). DNA was isolated as previously described (1–3). Sequencing was performed twice using the Ion PGM and Illumina MiSeq platforms. For the Ion PGM, 500-bp DNA fragments were prepared using the Ion PGM template IA 500 kit and sequenced using Hi-Q View chemistry on an Ion 316 Chip v2 (Thermo Fisher Scientific). For Illumina MiSeq 2 × 300-bp paired-end reads, the library was prepared using the Nextera XT DNA library prep kit.

The reads from a combined manifest file were assembled in MIRA v4.9.5_2 (4) using default parameters. The genomes were separated *in silico* in MaxBin v2.2.4 (5) using reads as input; the *Leptolyngbya* sp. genome was 96.3% complete, and the *Porphyrobacter* sp. genome was 97.2% complete. The genomes were annotated using the automated NCBI Prokaryotic Genome Annotation Pipeline (PGAP) (6, 7).

The draft genomic assembly of the *Leptolyngbya* sp. consisted of 187 scaffolds, an *N*_50_ value of 1.5 × 10^5^ nucleotides (nt), and a total size of 8.2 Mbp with an average read coverage of 65×. This genome contained 7,204 genes, with 6,725 coding DNA sequences (CDSs) and 81 RNAs. For the *Porphyrobacter* sp., the assembly consisted of 9 scaffolds, an *N*_50_ value of 1.1 × 10^6^ nt, and a total size of 3.5 Mbp with an average read coverage of 50×. The *Porphyrobacter* genome contained 3,327 genes, with 3,197 CDSs and 51 RNAs.

**FIG 1:**
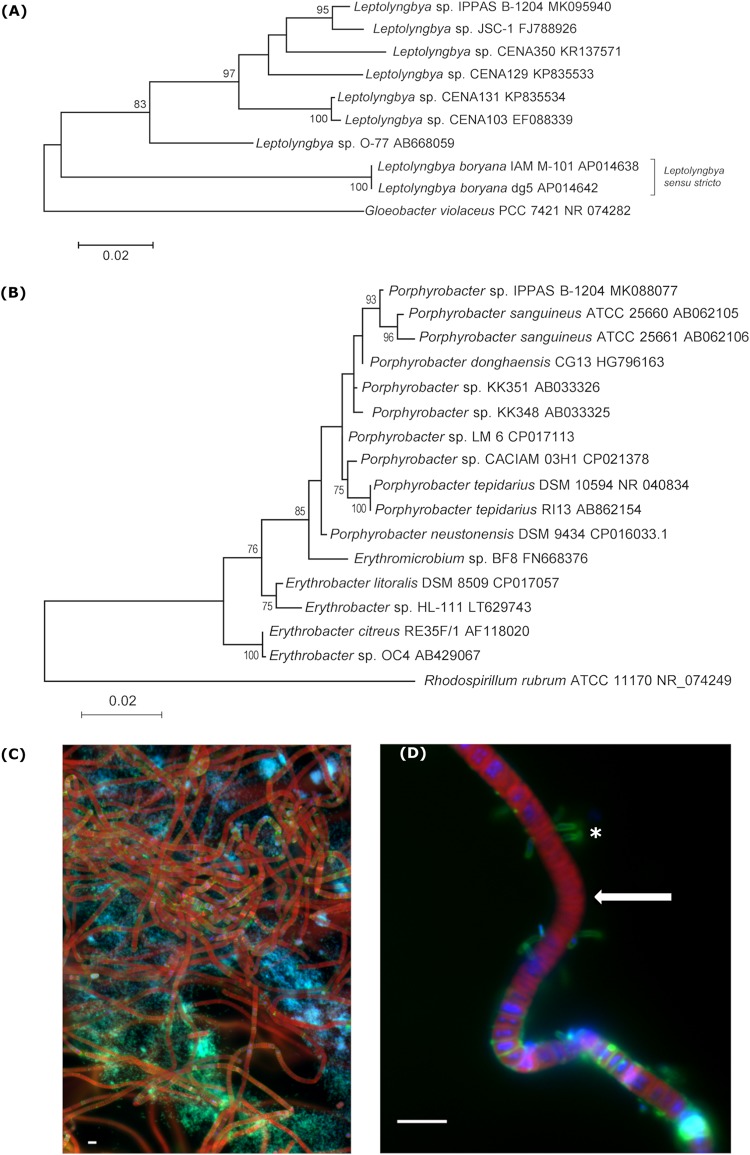
Putative prokaryotic consortium IPPAS B-1204. 16S rRNA gene phylogeny of the sequenced strains and microscopic images are shown. (A) 16S rRNA gene phylogeny of *Leptolyngbya* sp. strain IPPAS B-1204. (B) 16S rRNA gene phylogeny of *Porphyrobacter* sp. strain IPPAS B-1204. Phylogenetic analysis based on 16S rRNA sequences extracted manually after PGAP annotation was conducted using the maximum likelihood method based on the Kimura 2-parameter model (15) in MEGA7 (16). Initial trees for the heuristic search were obtained automatically by applying the neighbor-join and BioNJ algorithms to a matrix of pairwise distances estimated using the maximum composite likelihood (MCL) approach and then selecting the topology with the superior log likelihood value. Bootstrap values of >60% are shown. Gloeobacter violaceus PCC 7421 and Rhodospirillum rubrum ATCC 11170 were used as outgroups for panels A and B, respectively. (C) Merged fluorescence image of the IPPAS B-1204 culture stained with DAPI (4′,6-diamidino-2-phenylindole) for DNA (blue), FM 1-43 for cellular membranes (green), and autofluorescence of chlorophyll (red). Bar = 5 μm. (D) Magnified filament of the *Leptolyngbya* sp. (the arrow indicates trichomes visualized by blue and red) closely interacting with the *Porphyrobacter* sp. (asterisk indicates individual cells outlined by membranes stained green). Bar = 5 μm. Images were acquired using three channels of an Axio Imager Z2 epifluorescence microscope equipped with an AxioCam MRm high-resolution monochrome charge-coupled-device (CCD) camera and merged using AxioVision v4.8 software (Carl Zeiss, Göttingen, Germany). For the first channel, filter set 49 was used (excitation G 365, emission BP 445/50), and epifluorescence images of DAPI-DNA complexes were assigned a blue pseudocolor; for the second channel, filter set 44 was used (excitation BP 475/40, emission BP 530/50), and images of cell membranes stained with FM 1-43 were assigned a green pseudocolor; for the third channel, filter set 45 was used (excitation BP 560/40, emission BP 630/75), and cyanobacterial chlorophyll autofluorescence was assigned a red pseudocolor. Scale bar = 5 μm.

Phylogenetic analysis of 16S rRNA from the two IPPAS B-1204 genomes clustered them with two species, *Leptolyngbya* sp. strain JSC-1 (Fig. 1A) and Porphyrobacter sanguineus (Fig. 1B) with reliable bootstrap support. *Leptolyngbya* is a thermotolerant siderophilic cyanobacterium with chlorophylls *a*, *d*, and *f* and unusual carotenoids (8, 9), while *Porphyrobacter* is an aerobic chemooroganotrophic alphaproteobacterium (Fig. 1B). It is not unusual for *Porphyrobacter* spp. to be associated with cyanobacteria (10–12).

The genome of the *Leptolyngbya* sp. was analyzed with antiSMASH, which located gene clusters for biosynthesis of unusual carotenoids, alkaloids, antibiotics, the molluscicidal agent barbamide, nostopeptolide, nostophycin, yersiniabactin, lasso peptides, and nitrogen fixation.

The assumption that we were working with a consortium rather than two separate species in the same culture was supported by preliminary evidence similar to that described in reference 13. First, we were unable to isolate the axenic cyanobacterial component. Second, we found that the *Leptolyngbya* sp. negatively affected the growth of its partner, suggesting antibiosis. We also demonstrated a significant spatial proximity of the *Leptolyngbya* sp. and the *Porphyrobacter* sp. in IPPAS B-1204 (Fig. 1C and D), which implies putative trophic and biochemical interactions between the species. We are going to conduct a detailed polyphasic analysis (14) of these coexisting microorganisms in the future.

### Data availability.

The metagenome sequences are deposited in NCBI under BioProject number PRJNA498307, SRA project number SRP183214, and BioSample number SAMN10320329. The assembled genome of *Leptolyngbya* sp. IPPAS B-1204 is deposited under GenBank accession number RHGL00000000, and that of *Porphyrobacter* sp. IPPAS B-1204 under GenBank accession number RHGM00000000.
